# Prognostic factors associated with survival and hospitalization time in pediatric canine patients diagnosed with presumptive acute viral gastroenteritis

**DOI:** 10.14202/vetworld.2022.2095-2101

**Published:** 2022-08-30

**Authors:** Tomás Rodrigues Magalhães, Hugo Gregório, João Araújo, Lénio Ribeiro, Maria João Dourado, Sofia Batista, Felisbina Luisa Queiroga

**Affiliations:** 1 Department of Veterinary Sciences, University of Trás-os-Montes and Alto Douro, Vila Real, Portugal; 2 Animal and Veterinary Research Centre (CECAV), University of Trás-os-Montes and Alto Douro, Vila Real, Portugal; 3 Anicura - Centro Hospitalar Veterinário, R. Manuel Pinto de Azevedo 118 4100-320 Porto, Portugal; 4 Hospital Veterinário Bom Jesus, Av. General Carilho da Silva Pinto 52 4715-380 Braga, Portugal; 5 Center for the Study of Animal Sciences, CECA-ICETA, University of Porto, Porto, Portugal

**Keywords:** acute viral gastroenteritis, dog, infectious gastroenteritis, plasma administration, prognosis

## Abstract

**Background and Aim:**

Acute viral gastroenteritis is one of the main causes of hospitalization in dogs during the 1^st^ year of life. This retrospective study aimed to describe a pediatric canine population presumptively diagnosed with acute viral gastroenteritis and to identify potential prognostic factors that influence hospitalization time (HT) and mortality.

**Materials and Methods:**

Canine patients up to 12 months of age diagnosed with presumptive acute viral gastroenteritis were searched retrospectively from two veterinary hospitals during a 5-year period (2015–2020). Information regarding patient signalment, prophylactic care, clinical signs, blood test results, presence of systemic inflammatory response syndrome, and additional treatments were recorded to analyze their association with HT and mortality. Only dogs with a complete medical record until death or discharge were included in the study.

**Results:**

Ninety-four dogs were identified: 76 dogs (80.9%) survived with a median HT of 5 days (range: 2–16 days) and 18 dogs (19.1%) died with a median HT of 3½ days (range: 1–8 days) after admission. The presence of fever and fresh frozen plasma (FFP) administration was significantly associated with a lower survival rate (p = 0.021 and p = 0.037) in the multivariate analysis. Among survivors, incomplete primo-vaccination, the presence of hematochezia, and FFP administration were considered independent predictors of time to clinical recovery (p = 0.026, p = 0.047, and p = 0.026, respectively), being associated with higher HT.

**Conclusion:**

The presence of fever and FFP administration was significantly associated with a lower survival rate. An inadequate primo-vaccination status prior to admission, hematochezia, and FFP administration was associated with longer HT in surviving patients. Further studies are needed to confirm the present results.

## Introduction

Canine viral gastroenteritis represents a group of diseases characterized by viral-induced gastrointestinal inflammation [1]. Although 11 viral species have been identified in association with canine gastroenteritis, only four of them have been proven to cause this condition: Canine parvovirus (CPV), canine distemper virus (CDV), canine rotavirus, and canine enteric coronavirus [2].

Canine parvovirus is the most frequently diagnosed pathogen, causing the destruction of rapidly dividing cells present in intestinal crypts and lymphoid organs, leading to the manifestation of acute-onset signs of vomiting, diarrhea, lethargy, and anorexia [3]. Animals affected with viral gastroenteritis, particularly CPV, often have to be stabilized with inpatient therapy to treat life-threatening complications, such as hypovolemia, hypoalbuminemia, hypoglycemia, neutropenia, and sepsis [3]. Sepsis and systemic inflammatory response syndrome (SIRS) have also been diagnosed in critically ill dogs affected by viral gastroenteritis [4, 5].

To prevent viral infection, at-risk puppies must be kept protected from possible exposure until they complete a course of vaccinations from 6–8 weeks through 16 weeks of age or older, according to the latest World Small Animal Veterinary Association vaccination guidelines [6].

The standard treatment for viral gastroenteritis includes fluid therapy, broad-spectrum antibiotics, antiemetics, gastroprotective drugs, and interventional nutrition [3, 7]. Fresh frozen plasma (FFP) administration may also benefit patients with CPV [8], although its use was considered unclear in critically ill animals in a previous study [9]. Additional therapies such as the use of feline anti-parvovirus antibodies [10], paraimunity inducers derived from poxviruses [11], a human immunomodulator [12], and fecal microbiota transplantation [13] have also been investigated, with only the last two showing therapeutic benefit so far.

The prognosis of dogs with viral gastroenteritis has already been associated with the severity of clinical signs and laboratory abnormalities at the time of diagnosis, the presence of comorbidities, and the therapeutic approach elected [3], but further studies are needed to identify more potential prognostic factors. Moreover, associated long-term effects have been proposed as surviving patients appear to be more predisposed to the development of chronic gastrointestinal disease [14, 15].

This retrospective study aimed to describe a pediatric canine population presumptively diagnosed with acute viral gastroenteritis and to identify potential prognostic factors that influence hospitalization time (HT) and mortality.

## Material and Methods

### Ethical approval

Because of the retrospective nature of this study and the use of diagnostic data collected as a part of routine clinical procedures, the need for ethical approval was waived.

### Study period and location

The archived medical records (from January 2015 to December 2020) of two referral veterinary hospitals in the northern region of Portugal were searched for juvenile dogs diagnosed and treated for acute gastroenteritis presumed to be viral in origin.

### Inclusion criteria

Inclusion criteria were patients diagnosed with presumptive acute viral gastroenteritis up to 12 months of age and with a complete medical record until the time of discharge or death. Dogs were excluded if the cause of gastroenteritis was related to toxin ingestion, gastrointestinal foreign bodies, sudden change in diet, recent medication, or even infectious agents other than viruses (e.g., bacteria, fungi, or parasites). Moreover, patients were excluded if they had an incomplete medical history or an inadequate follow-up due to insufficient data regarding their clinical case or outcome.

For each dog, patient information regarding signalment, such as age, sex, weight, breed and size, prophylactic care, including deworming and vaccination status, specific clinical signs, in particular hematochezia and fever, blood test results, namely, nadir neutrophil count and serum albumin concentration, and additional treatments, such as synthetic colloids infusion and FFP administration, were recorded. The presence of SIRS at admission was also evaluated, and the diagnosis was made based on the criteria previously applied in CPV patients [5], which presupposes the manifestation of at least three of the four following conditions: A heart rate >140 beats/min; a respiratory rate >30 breaths/min; a body temperature above 39.2°C or below 37.8°C; and a white blood cell count >17,000 or <6000 cells/mL. The association between these parameters and HT and mortality was analyzed. The HT was measured, in days, from admission to clinical discharge or death, on the surviving and non-surviving dogs, respectively.

### Statistical analysis

The IBM^â^ SPSS^â^ Statistics Software (Version 27.0; IBM Corp^ã^, Armonk, NY, USA) was used for the statistical analysis of results. Categorical variables were expressed in absolute and relative frequencies, and for the continuous variables, median and respective range were also measured.

The association between the clinical variables and outcome (surviving vs. non-surviving) was calculated by the Chi-square test and Fisher’s exact test. For this purpose, continuous variables, such as age, weight, and nadir neutrophil count, were transformed into categorical ones. All variables that showed a significant association (p < 0.05) with the outcome in the univariate analysis were studied using a multivariable logistic regression model to determine whether they could be considered independent prognostic factors.

Median, mean, and standard deviation were calculated for the HT. Shapiro–Wilk test of normality was performed and confirmed that HT did not follow the normal distribution. Therefore, Mann–Whitney U and Kruskal–Wallis H tests were carried out to assess their association with the clinical variables. As performed for the outcome, a multivariate analysis was also carried out with the variables that previously showed a statistically significant association with the HT (p < 0.05), using the Cox proportional hazards for this purpose.

## Results

In this study, 112 dogs were initially identified, but 18 were eventually excluded due to age above the defined limit (n = 1), loss to follow-up (n = 4), uncertainty about the vaccination status (n = 12), and the outcome (n = 1). Therefore, 94 dogs met the final inclusion criteria and were considered for this retrospective study.

### Patient signalment

In our canine population, 52.1% (n = 49) were male and 47.9% (n = 45) were female. The median age was 16 weeks (range: 4–52 weeks) and the median weight at diagnosis was 6.76 kg (range: 0.6–41.4 kg). In terms of dog breed, 48 dogs were purebred and 46 were crossbred. The most frequent purebreds were Labrador (n = 8), Pinscher (n = 5), Estrela Mountain dog (n = 4), German Shepherd (n = 4), and Rafeiro do Alentejo (n = 3), in addition to four breeds with only two dogs each (Border Collie, Cane Corso, Spanish Mastiff, and Swiss Shepherd dog) and to 16 breeds represented by only one patient (Beagle, Boerboel, Boxer, Bull Terrier, Dalmatian, Dobermann, Dutch Shepherd dog, English Bulldog, English Cocker Spaniel, Epagneul Breton, German Spitz, Great Dane, Miniature Poodle, Pitbull, Transmontano Cattle dog, and Yorkshire terrier). For purebred dogs, 18.8% (n = 9) were small sized, 16.7% (n = 8) were medium sized, and 64.6% (n = 31) were large sized, according to the expected adult size and the breed standard.

### Prophylactic care

According to prophylactic status, 75.5% (n = 71) had not received any vaccine, 20.2% (n = 19) had been vaccinated, but the initial protocol was still incomplete, and 4.3% (n = 4) had already finished the primo-vaccination schedule at the time of diagnosis.

Information about the deworming protocol was available on 84 animals. An adequate deworming schedule was considered when it started at 2 weeks of age and was carried out fortnightly at first and then monthly for year-round control, based on the 2019 AAHA Canine Life Stage Guidelines [16]. Considering this, only 37 dogs were considered to have the aforementioned scheme correctly performed at the time they were admitted to the hospital. Nevertheless, it should be noted that the administration of internal deworming was always performed after admission to eliminate possible intestinal parasites and thus rule out parasitic causes.

### Clinical signs and blood test results

Specific clinical signs and blood test results considered of interest for the study were retrieved. Hematochezia was present in most of the dogs included: 71.3% (n = 67). Fever was present in half of the dogs (n = 47) and only 33.0% (n = 31) showed signs related to SIRS at presentation. Serum albumin concentration at admission was available in only 72 patients, 34 of whom had hypoalbuminemia (reference range: 2.7–3.6 g/dL). The median nadir neutrophil count was 1.35 × 10^9^/L with a range from 0.06 to 12.9 × 10^9^/L.

### Diagnosis and treatment

All dogs included were diagnosed with acute viral gastroenteritis, either by a positive fecal antigen test for one of the aforementioned viruses (preferably) or through clinical reasoning, when financial constraints prevented its performance. In the latter cases, only animals that had a strong clinical suspicion toward a viral agent were included, given the clinical presentation, laboratory findings, and response to treatment, in addition to evidence of performing ancillary tests that ruled out other potential causes. It should also be noted that all cases had a clinical diagnosis of CPV enteritis, but as no enzyme-linked immunosorbent assay or polymerase chain reaction test was performed for confirmation in all 94 animals, we chose a more general term, admitting the possibility of a misdiagnosis. In fact, although unlikely, considering the clinical presentation, CDV, enteric coronavirus, or rotavirus may have been responsible for gastroenteritis in some patients, either primarily or in coinfection. In any case, as mentioned, the possibility that the etiology was non-viral was ruled out based on anamnesis and clinical and laboratory findings obtained through physical examination and complementary diagnostic tests (e.g., fecal testing).

Therefore, the general symptomatic treatment of acute viral gastroenteritis was provided for all patients: Intravenous crystalloid fluid therapy, broad-spectrum antibiotics, antiemetic and gastroprotective medication, and interventional nutrition. In some cases, additional therapies were also administered: 7.4% (n = 7) received synthetic colloids infusion and 26.6% (n = 25) received FFP administration. The median total plasma dose provided was 10 mL/kg with a range from 4.7 to 50 mL/kg. No adverse reactions were described in any of the transfused patients.

Patients were kept hospitalized until clinical recovery and were discharged when vomiting and diarrhea had stopped, the appetite had returned to normal and the blood parameters such as packed cell volume, neutrophil count, and albumin normalized.

### Hospitalization time and survival

The survival rate was 80.9%: 76 dogs survived and were discharged, but 18 dogs (19.1%) died. Among the patients that died, two were euthanized due to poor prognosis. The median hospitalized time for patients that died was 3½ days (range: 1–8 days). For the surviving patients, the median HT was 5 days (range: 2–16 days).

A nadir neutrophil count lower than 1.35 × 10^9^/L, the presence of fever, and the need for FFP administration were significantly associated with lower survival rate (p = 0.017, p = 0.017, and p = 0.006, respectively) in the univariate analysis, but only the last two showed a significant association with the outcome in the multivariate analysis (p = 0.021 and p = 0.037) (Table-1).

**Table-1:** Association between patient signalment, clinical presentation, and treatment with outcome (surviving vs. non-surviving patients) in 94 dogs diagnosed with presumptive acute viral gastroenteritis from two referral hospitals in the northern region of Portugal.

Variables	Surviving patients (n)	Non-surviving patients (n)	Survival rate (%)	Univariate analysis	Multivariate analysis	
	
p-value				p-value	HR + (95% CI)
Age (n = 94)				0.066	—	—
<16 weeks (n = 43)	31	12	72.1			
≥16 weeks (n = 51)	45	6	88.2			
Sex (n = 94)				0.601	—	—
Male (n = 49)	41	8	83.7			
Female (n = 45)	35	10	77.8			
Weight (n = 94)				0.189	—	—
<6.76 kg (n = 47)	35	12	74.5			
≥6.76 kg (n = 47)	41	6	87.2			
Breed (n = 94)				0.605	—	—
Purebred (n = 48)	40	8	83.3			
Crossbred (n = 46)	36	10	78.3			
Deworming protocol (n = 84)				0.585	—	—
Adequate (n = 37)	31	6	83.8			
Inadequate (n = 47)	36	11	76.6			
Primo-vaccination status (n = 94)				0.303	—	—
Complete (n = 4)	4	0	100.0			
Incomplete (n = 19)	17	2	89.5			
Not performed (n = 71)	55	16	77.5			
Hematochezia (n = 94)				0.576	—	—
Present (n = 67)	53	14	79.1			
Absent (n = 27)	23	4	85.2			
Fever (n = 94)				**0.017**	**0.021**	4.46 (1.25–15.90)
Present (n = 47)	33	14	70.2			
Absent (n = 47)	43	4	91.5			
SIRS (n = 94)				0.584	—	—
Present (n = 31)	24	7	77.4			
Absent (n = 63)	52	11	82.5			
Hypoalbuminemia (n = 72)				1.000	—	—
Present (n = 34)	26	8	76.5			
Absent (n = 38)	30	8	78.9			
Nadir neutrophil count (n = 94)				**0.017**	0.125	0.36 (0.10 – 1.33)
<1.35×10^9^/L (n = 47)	33	14	70.2			
≥1.35×10^9^/L (n = 47)	43	4	91.5			
FFP administration (n = 94)				**0.006**	**0.037**	3.67 (1.08 – 12.43)
Performed (n = 25)	15	10	60.0			
Not performed (n = 69)	61	8	88.4			

CI=Confidence interval, FFP=Fresh frozen plasma, HR=Hazard ratio, n=Number of patients, P=Level of significance, %=Percentage, SIRS=Systemic inflammatory response syndrome. All statistically significant associations have the p-value in bold

The impact of different variables on the HT of dogs that survived was evaluated. One dog of the 76 survivors was excluded because the exact duration of its hospitalization could not be ascertained, although it was confirmed that this patient survived and was discharged from the hospital. Therefore, 75 dogs were evaluated at this stage. In the univariate analysis, HT showed a statistically significant association with the primo-vaccination status, presence of hematochezia, nadir neutrophil count, and plasma administration (p = 0.029, p = 0.032, p = 0.041, and p = 0.001, respectively) (Table-2). Following multivariate analysis, it was concluded that patients with inadequate vaccination status, hematochezia, and that underwent FFP administration were associated with longer HTs (p = 0.026, p = 0.047, and p = 0.026, respectively).

**Table-2:** Association between patient signalment, clinical presentation, and treatment with HT in the 75 surviving dogs that clinically recovered from presumptive acute viral gastroenteritis.

Variables	n	Median HT	Mean HT	SE	Univariate analysis	Multivariate analysis	
	
p-value					p-value	HR + (95% CI)
Age (n = 75)					0.645	—	—
<16 weeks	31	5.00	5.52	2.72			
≥16 weeks	44	5.00	5.89	3.09			
Sex (n = 75)					0.360	—	—
Male	40	5.50	6.00	2.97			
Female	35	5.00	5.43	2.89			
Weight (n = 75)					0.429	—	—
<6.76 kg	35	5.00	5.46	2.93			
≥6.76 kg	40	5.00	5.98	2.93			
Breed (n = 75)					0.630	—	—
Purebred	40	5.50	5.93	3.10			
Crossbred	35	5.00	5.51	2.75			
Deworming protocol (n = 66)					0.484	—	—
Adequate	30	6.50	6.13	3.09			
Inadequate	36	5.00	5.64	2.97			
Primo-vaccination status (n = 75)					**0.029**	**0.026**	3.87 (1.18–12.77)
Complete	4	3.00	2.75	0.50			
Incomplete Not performed	17	5.00	5.24	2.84			
Not performed	54	5.00	6.11	2.94			
Hematochezia (n = 75)					**0.032**	**0.047**	1.74 (1.01–3.00)
Present	52	5.50	6.17	2.97			
Absent	23	5.00	4.74	2.63			
Fever (n = 75)					0.460	—	—
Present	32	5.00	6.13	3.32			
Absent	43	5.00	5.44	2.60			
sirs (n = 75)					0.954	—	—
Present	23	5.00	5.65	2.67			
Absent	52	5.00	5.77	3.06			
Hypoalbuminemia (n = 55)					0.380	—	—
Present	25	6.00	6.32	3.54			
Absent	30	5.00	5.37	2.37			
Nadir neutrophil count (n = 75)					**0.041**	0.097	0.65 (0.40–1.08)
<1.35×10^9^/L	33	6.00	6.58	3.24			
≥1.35×10^9^/L	42	5.00	5.07	2.50			
FFP administration (n = 75)					**0.001**	**0.026**	2.05 (1.09–3.87)
Performed	15	8.00	8.20	3.21			
Not performed	60	5.00	5.12	2.53			

CI=Confidence interval, FFP=Fresh frozen plasma, HR=Hazard ratio, n=Number of patients, p=Level of significance, SE=Standard error, %=Percentage, SIRS=Systemic inflammatory response syndrome, HT=Hospitalization time.

All statistically significant associations have the p-value in bold

## Discussion

The present study retrospectively evaluated a group of young dogs diagnosed with acute presumptive viral gastroenteritis. The main objective was to assess potential prognostic factors associated with HT and survival and to describe this pediatric canine population.

The signalment of affected animals was very similar to other recently studied canine populations diagnosed with acute viral gastroenteritis, such as CPV enteritis [4, 5, 17]. In general, survival rate and HT were comparable with the results of the previous studies [5, 10, 13, 18].

The majority of the population was represented by puppies under 6 months of age with an inadequate vaccination protocol. In fact, patients with an inadequate primo-vaccination status needed significantly more time to clinically recover (p = 0.026), probably as a result of a less competent immune response.

Hematochezia was one of the most frequent clinical signs observed in affected animals (71.3%). This was an interesting finding because although diarrhea remains one of the most reported signs in patients with viral gastroenteritis, the presence of blood has been described in a lower percentage of patients (21–51%) [5, 14, 17]. Patients with hematochezia at admission had significantly longer HT (p = 0.047), probably because that condition was associated with a more severe degree of enteritis that required more time for its stabilization.

Fever was present in exactly half of the animals (50%), at the time of presentation, which is a higher relative percentage compared to the previous studies, such as Kalli *et al*. [5] and Chalifoux *et al*. [17], who reported pyrexia in only 33% and 26% of their patients, respectively. The survival rate was significantly lower in the febrile patient group compared to the group of patients without fever at presentation (70.2% vs. 91.5%). This significant association with the outcome was confirmed when the multivariate analysis model was applied. Interestingly, in a previous study, Kalli *et al*. [5] described a different association that patients with fever at admission had 16.6 times higher odds of surviving, an unexpected finding that the authors could not explain [5]. Further prospective studies dividing animals into two groups (febrile vs. non-febrile patients) are needed to assess its true prognostic effect.

One reason for the differences observed in clinical presentation may be related to the stage of the disease at the time of admission: Possibly, our patients were hospitalized later in the course of the disease, allowing signs such as hematochezia to be already present. Nevertheless, this assumption was not possible to prove because the median duration of presenting complaints before admission could not be calculated for further comparisons. Even so, only 33.0% of our patients showed signs compatible with SIRS, which is in line with the frequency of this condition described by other authors based on the same criteria [5].

As described in previous studies [5, 17], hypoalbuminemia and neutropenia were the most common laboratory abnormalities in this study population. The association between neutropenic CPV patients and a negative prognosis was previously described by Venn *et al*. [18] in a prospective study in which all non-surviving dogs were neutropenic at the time of death. Our study observed the same tendency, with animals with lower neutrophil nadir count showing the lowest survival rate. However, this significance was lost in the multivariate analysis model. It should be noted that although they have not been studied in the present work, other laboratory parameters appear to be related to the course and prognosis of the disease, such as hematocrit [17] and mean platelet volume and platelet volume distribution [19], as recently published. Further studies will be needed to assess these potential prognostic associations.

In addition to the supportive treatment provided to all patients, other therapies were described. The FFP was administered in 25 patients, mainly to correct hypoalbuminemia, as previously described [8]. Our study failed to prove its clinical benefit, as FFP administration was associated with a lower survival rate and a longer HT, and different aspects may have contributed to this result. One of the major reasons is related to the fact that this therapeutic intervention is still not performed by routine but held only for complicated cases with a very poor clinical conditions. Moreover, the dose administered could have compromised its effect since the median plasma dose prescribed (10 mL/kg) may have been insufficient to produce a significant rise in serum albumin concentration, as reported by other authors with even higher median doses (15–18 mL/kg) [20]. In fact, to increase serum albumin concentration by 0.5 g/dL, it is required at least 25 mL/kg of FFP [21]. The financial costs associated with this therapy limit the use of higher doses of FFP [20, 22]. Alternative products with higher albumin concentrations, such as cryoprecipitate-poor plasma, concentrated human, or canine albumin products, may be less expensive and more effective [21, 23]. Although reactions such as fever, pruritus, and anxiety have been described in a small percentage of transfused patients [20, 22], no side effects were reported in this study, which suggests that this therapeutic approach is safe and well tolerated in critically ill patients diagnosed with presumptive infectious gastroenteritis. This finding is in line with a recent publication [22]. Our study makes clear the non-therapeutic effect of this FFP dosage and advocates for further studies to clarify the present results. Another additional therapy described was synthetic colloid infusion, but the number of patients undergoing it was so small (n = 7) that it precluded consequent analysis.

### Limitations of the study

Limitations of this study include its retrospective nature, the subjective conclusion of viral cause of gastroenteritis, heterogeneity of the patient population, variations in the treatment protocol provided, and incomplete reporting on clinical files. The potential impact of these study weaknesses was minimized by choosing only two referral veterinary hospitals with very similar clinical protocols and work practices and by including only patients with available clinical data and with a clear and known outcome (clinical discharge or death).

Another limitation is the diagnostic approach since not all animals performed antigen tests for detection of viruses, such as CPV and CDV. Our diagnosis was mostly clinical, considering the clinical signs, the laboratory abnormalities, the course of the disease, and the treatment response, in addition to the exclusion of the respective differential diagnoses. The treatment of CPV and CDV in young dogs is often expensive for the newly pet owners due to the need of hospitalization for an extended period of time, the aggressive multimodal therapy required, and the regular clinical and laboratory monitoring. For that same reason, an outpatient protocol has already been described for situations where hospitalization cannot be supported by the owner [18]. Therefore, clinicians often have to make choices to treat their patients, considering the financial restrictions of their clients. To that end, parvoviral enteritis is frequently diagnosed through clinical reasoning in our country, rather than performing confirmatory fecal antigen testing. Clinical reasoning is an essential skill to the process of decision-making in everyday clinical practice, based on past experience and deduction methods, as it has been explored [24, 25].

## Conclusion

This study illustrates the current state of presumptive viral gastroenteritis and provides new information regarding the impact of various epidemiological, clinical, and therapeutic features on the prognosis. Our main conclusions are: (1) The majority of dogs diagnosed with acute viral gastroenteritis showed an inadequate vaccination status and severe clinical and laboratory abnormalities upon admission; (2) the presence of fever was significantly associated with a lower survival rate; (3) an inadequate primo-vaccination protocol prior admission and hematochezia was significantly associated with longer HT for patients that survived; and (4) the FFP administration in the regimen performed in this subgroup of patients was associated with worst prognosis, making its use in other conditions necessary for a better assessment of its potential therapeutic effect.

## Authors’ Contributions

HG, JA, and FLQ: Conception and design. JA, LR, MJD, and SB: Data collection. TRM, HG, and FLQ: Analysis and interpretation. TRM: Manuscript writing. TRM and FLQ: Reviewed and edited the manuscript. All authors have read and approved the final manuscript.
